# Psychic Life of Micro-Organisms

**Published:** 1889-06

**Authors:** 


THE PSYCHIC LIFE OF MIRRO-ORGANISMS.—A study in Experimental Psychology. By Alfred Binet. Translated from the French by Thomas McCormack, with a preface by the author written especially for the American edition. Chicago: 1889. The open Courts Publishing Company. Cloth, 75 cents. Paper, 50 cents.

M. Alfred Binet, the collaborator of Ribot and Feré, and one of the most eminent representatives of the French School of Psychology, has presented in the above work the most important results of recent investigations into the world of Micro-Organisms. The subject is a branch of compartive psychology little known, as the data of this department of natural science lie scattered for the most part in isolated reports and publications, and no attempt has hitherto been made to collate and present them in a systematised form.

M. Binet's researches and conclusions show, 'psychological phenomena begin among the very lowest classes of beings; they are met with in every form of life from the simplest cell to the most complicated organism." The author contests the theory of the distinguished English scientist, Prof. George J. Romanes, who assigns the first appearance of the various psychical and mental faculties to different stages or periods in the scale of zoological development. To. M. Binet there is an aggregate of properties which exclusively pertain to living matter, the existence of which is seen in the lowest forms of life as well as the highest.

